# Design and construction of a customizable phantom for the characterization of the three‐dimensional magnetic resonance imaging geometric distortion

**DOI:** 10.1002/acm2.13462

**Published:** 2021-10-31

**Authors:** Tarraf Torfeh, Rabih Hammoud, Satheesh Paloor, Yoganathan Arunachalam, Souha Aouadi, Noora Al‐Hammadi

**Affiliations:** ^1^ Department of Radiation Oncology National Center for Cancer Care and Research (NCCCR) Hamad Medical Corporation Doha Qatar

**Keywords:** 3D geometric distortion, customizable phantom, MR‐guided radiation therapy, MRI

## Abstract

One of the main challenges to using magnetic resonance imaging (MRI) in radiotherapy is the existence of system‐related geometric inaccuracies caused mainly by the inhomogeneity in the main magnetic field and the nonlinearities of the gradient coils.

Several physical phantoms, with fixed configuration, have been developed and commercialized for the assessment of the MRI geometric distortion. In this study, we propose a new design of a customizable phantom that can fit any type of radio frequency (RF) coil. It is composed of 3D printed plastic blocks containing holes that can hold glass tubes which can be filled with any liquid. The blocks can be assembled to construct phantoms with any dimension.

The feasibility of this design has been demonstrated by assembling four phantoms with high robustness allowing the assessment of the geometric distortion for the GE split head coil, the head and neck array coil, the anterior array coil, and the body coil. Phantom reproducibility was evaluated by analyzing the geometric distortion on CT acquisition of five independent assemblages of the phantom.

This solution meets all expectations in terms of having a robust, lightweight, modular, and practical tool for measuring distortion in three dimensions. Mean error in the position of the tubes was less than 0.2 mm. For the geometric distortion, our results showed that for all typical MRI sequences used for radiotherapy, the mean geometric distortion was less than 1 mm and less than 2.5 mm over radial distances of 150 mm and 250 mm, respectively.

These tools will be part of a quality assurance program aimed at monitoring the image quality of MRI scanners used to guide radiation therapy.

## 1 INTRODUCTION

Data from magnetic resonance imaging (MRI) are increasingly being used in the delineation of tumors and organs at risk (OAR) in the radiation therapy workflow, due mainly to its superior soft‐tissue contrast compared with computed tomography (CT).^1^, ^2^, ^3^

Furthermore, MRI‐only based dose planning for external beam radiation therapy has been increasingly gaining research interests and some commercial solutions have been already proposed and adopted.^4^, ^5^ The main reason behind this interest resides in the fact that the current planning of radiotherapy treatments that combines MRI and CT through image registration has several drawbacks such as the registration errors, the irradiation dose to the patient from CT, and the time allocated.

Finally, the clinical introduction of MRI‐guided radiotherapy with an MRI‐Linac^6^, ^7^ has allowed in addition to acquiring superior soft‐tissue images, the possibility to perform real‐time imaging during treatment as well as performing online adaptive planning.^8^, ^9^

However, one of the main challenges to using MRI in radiotherapy is the existence of system‐related geometric inaccuracies caused mainly by the inhomogeneity in the main magnetic field and the nonlinearities of the gradient coils. The assessment of system‐related geometric distortion has been extensively reported in the literature.^10^, ^11^, ^12^, ^13^, ^14^, ^15^, ^16^, ^17^, ^18^, ^19^, ^20^, ^21^ The dosimetric impact of this distortion has also been analyzed and reported.^22^, ^23^, ^24^, ^25^

Measurements carried out in these studies are based on phantoms with fixed configurations. Some of these phantoms were constructed in‐house^11^, ^14^, ^16^ while others were commercialized.^13^, ^17^ Some of these phantoms allowed the measurement of in‐plane distortion,^11^, ^13^, ^17^ while others permitted the measurement of in‐plane and through‐plane^10^, ^12^, ^16^ distortion. Furthermore, some of these phantoms covered a small field of view (FOV),^10^, ^13^ while others covered a large FOV.^11^, ^12^, ^14^, ^15^, ^16^, ^17^, ^19^, ^21^ Finally, some phantoms can be categorized as heavy since they are filled with water,^10^, ^11^, ^15^, ^20^, ^21^ while others are foam‐based or plastic‐based and thus lightweight phantoms.^12^, ^14^, ^16^, ^19^

The design of the phantom is crucial for an accurate, easy, and reproducible assessment of MRI distortion especially if it is used for RT. As such, it is very important to have a phantom covering a large FOV and allowing the measurement of the through plane distortion in addition to the in‐plane distortion. Finally, having a lightweight phantom that can be easily maneuvered is also essential. None of the previously developed phantoms meet all these requirements. Lately, a new study has been published^26^ proposing a modular phantom assembled from a series of rectangular foam blocks containing high‐contrast fiducials made of paintballs. The proposed design is very interesting as it allows assembling lightweight phantoms with different geometries that fit different coils.

In this study, we propose a novel design for a customizable phantom composed of three‐dimensional (3D) printed plastic blocks containing holes that can hold glass tubes. The plastic blocks can be assembled to construct phantoms with any dimension. The glass tubes spaced 25 mm can contain any liquid and are arranged in two directions, anterior/posterior and left/right, allowing the assessment of in‐plane and through‐plane distortion. In addition to providing the possibility of constructing customizable lightweight phantoms, the use of glass tubes instead of fiducials as control points presents several advantages including the possibility of filling any type of liquid. Furthermore, since the tubes cover the whole space perpendicular to the acquisition plane without any gap, a slice at any position will certainly intersect with the tubes. As such, for measuring the geometric distortion, an acquisition can be directly performed at the desired position with the certainty of having an image with signal coming from the tube. By contrast, for the phantoms using control points, the whole 3D volume should be acquired, and a reformatting should be done in order to produce images containing signal coming from the control points before measuring the geometric distortion.

## 2 MATERIALS AND METHODS

### 2.1 Phantom design

**FIGURE 1:**
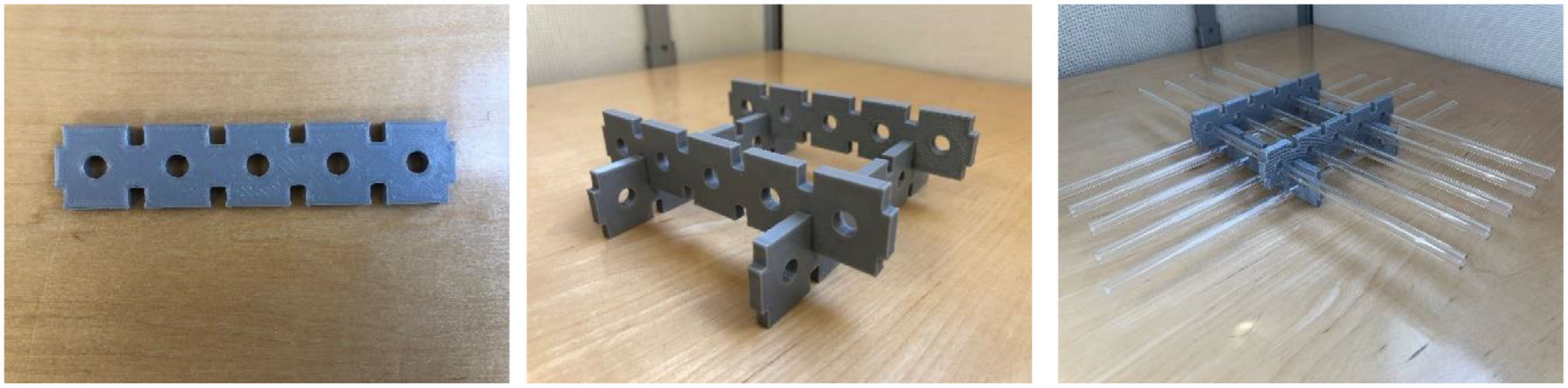
The design of the proposed phantom. (a) Plastic block with five holes, (b) four plastic blocks assembled together, and (c) ten glass tubes inserted into the holes

The phantom presented in this work is assembled from two types of 3D printed plastic blocks. The first type measures 75 × 25 × 5 mm^3^ and contains three holes of 7.1 mm diameter, while the second type measures 125 × 25 × 5 mm^3^ and containing five holes of 7.1 mm diameters each (Figure 1). The proposed design uses tongue and groove joints that allow these blocks to be easily assembled and to form any type of geometry. The holes inside the blocks allow holding glass tubes with an external diameter of 7 mm (Figure 1) that can be filled with any liquid. The plastic blocks used exhibit no MRI signal and allow a good contrast with the liquid inside the tubes.

**FIGURE 2:**
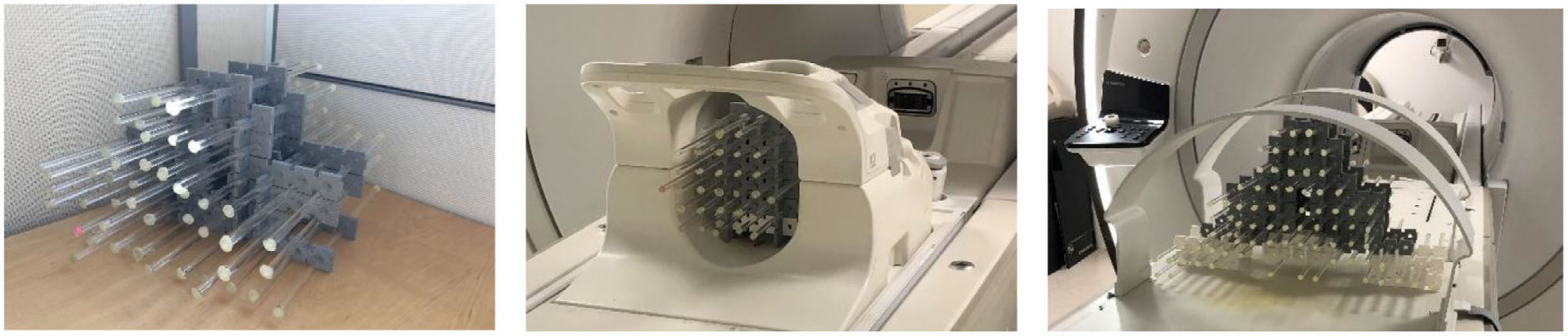
(a) The phantom made of assembled plastic blocks and glass tubes filled with water, (b) assembled phantom that fits the head coil, and (c) assembled phantom that fits the anterior coil

The proposed design allows building phantoms with any desired dimension and containing glass tubes arranged parallel to the lateral and the superior–inferior axes. These tubes are spaced 25 mm in each direction. In this study, these blocks along with glass tubes of 7 mm outer diameter and 5.5 mm inner diameter filled with water were used to assemble three phantoms. The first phantom of 500 × 450 × 300 mm^3^ was assembled using 160 blocks and used to assess the geometric distortion of the body coil. The second phantom of 350 × 350 × 300 mm^3^ was assembled using 100 blocks and used to assess the geometric distortion of the anterior coil. The third phantom of 200 × 200 × 30 mm^3^ was assembled using 60 blocks and used to assess the geometric distortion of the head coil as well as the head and the neck array coil. Figure 2(a) presents assembled plastic blocks with glass tubes filled with water. Figures 2(b), 2(c) present assembled phantoms that fit the head coil and anterior array coil, respectively.

### 2.2 Reproducibility

The accuracy of reproducibility of the phantom was evaluated by analyzing the positioning error of the tubes using CT acquisition of the phantoms. For this, the 160 blocks forming the largest phantom were dissembled and reassembled. This procedure was repeated five times and the geometric distortion for all acquisitions was measured and compared.

### 2.3 Clinical MRI geometric distortion

MRI image acquisition was performed on a GE 1.5T MRI‐SIM,450 W unit commissioned for RT planning. The geometric distortion was assessed for the GE split head coil, the head and neck array coil, the anterior array coil, and the body coil.

For the GE split head coil, axial T1 spin echo, axial T2 spin echo, and 3D axial cube sequences were used. Two sequences were used for the head and neck array coil; axial T1 spin echo and axial T2 spin echo. For the anterior array coil, 3D axial T1 and 3D sagittal T2 sequences were studied. Finally, T1 fast spin echo, 3D axial cube T1, 3D axial cube T2, 3D sagittal cube T1, 3D sagittal cube T2, 3D coronal cube T1, and 3D coronal cube T2 were used for the body coil. These sequences, which are summarized in Table 1, are used for RT applications in our department.

**TABLE 1:** MRI sequences used in this study

		**Acquisition details**			
**Coil**	**Series**	**TR (ms)**	**TE (ms)**	**ETL**	**Pixel Bandwidth (Hz)**
Body coil	Axial Cube T1	600	103	24	244
	Axial Cube T2	2000	99	100	244
	Sagittal Cube T1	600	10.3	24	488
	Sagittal Cube T2	2000	104	90	390
	Coronal Cube T1	600	10.3	23	244
	Coronal Cube T2	600	114	100	244
Anterior array coil	3D axial T1	7.2	1.9	1	244
	3D sagittal T2	2240	97	100	162
Head and Neck array coil	Axial T1 spin echo	500	20	1	122
	Axial T2 spin echo	2000	20	1	122
Head coil	Axial T2 Cube	1300	20	50	244
	Axial T1 spin echo	534	7.4	4	139
	Axial T2 fast spin echo	2500	99	18	139

**FIGURE 3:**
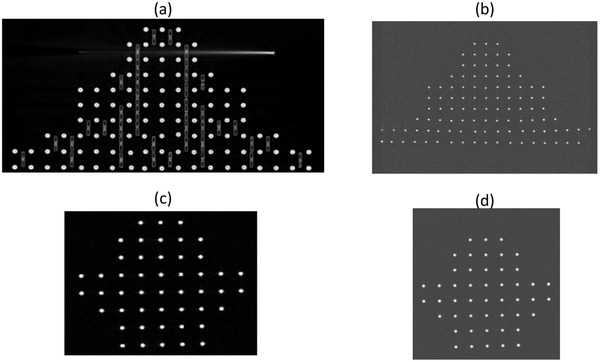
Axial CT and MRI slices of the phantom. (a) and (b) CT and MRI slices of the phantom used for the body coil. (c) and (d) CT and MRI slices of the phantom used for the head coil

CT dataset of the phantom was first performed using Siemens Somatom Sensation CT simulator. For this, 350 slices of 1 mm thickness with no gap, a FOV of 500 mm and a pixel size of 0.97 mm were acquired. This dataset will create a gold standard spatial representation of the tubes against which MRI images will be compared (Figure 3).

### 2.4 Image analysis

**FIGURE 4:**
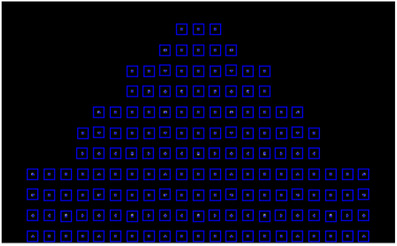
Detecting process showing the square regions and the detected tubes

Java‐based software for automating the process of measuring the geometric distortion is developed based on the same algorithms that we have used in our previous work.^11^, ^12^ The center of each image is first calculated by projecting the pixels vertically and horizontally and the center of each projection represents the center of the image. A square region is then constructed around the central tube and the same projection method is used to calculate center of the tube inside this region and adjust the position of the square region accordingly. Square regions surrounding adjacent tubes are then constructed and their position adjusted using the same method. The process is repeated in order to have a square region for every tube. Finally, the exact position of the center of each tube is calculated by producing a binary image for each region based on threshold defined by this equation:

(1) $$\mathrm{threshold}\;=\;\mathrm{bg}+\left(\max-\mathrm{bg}\right)\times 0.5,$$

where bg is the mean intensity value of the background and max is the maximum intensity value inside the region. The coordinates of each tube are then calculated from the center of mass of the pixels inside the binary region as shown in Figure 4.

3D maps representing the positions of the tubes are then produced on CT and MRI dataset. Finally, the rigid registration of the MRI and the reference‐CT images is then performed using the same method that was used in our previous work.^11^ In this method, phantom misalignment errors (translational and rotational) are automatically corrected by minimizing the distance error between position of the tubes calculated on CT and their corresponding on MRI. The geometric distortion is then calculated as the difference between the coordinates of each corresponding MRI and CT position.

### 2.5 Software validation

**FIGURE 5:**
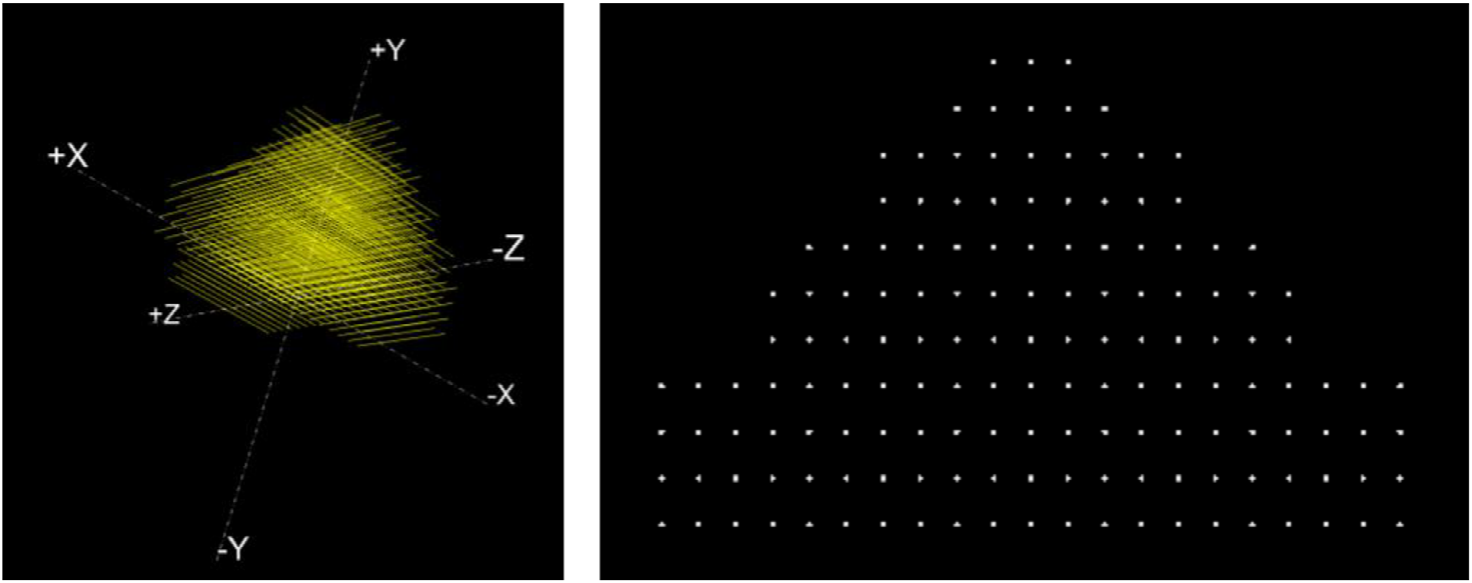
(a) 3D virtual phantom and (b) 2D image of the phantom

The software was validated using synthetic images based on the same methodology that was used in our previous work.^11^ Three virtual phantoms were created reproducing the geometry of our three physical phantoms. Known amount of spatial distortion is then introduced to these virtual phantoms in three directions. Finally, synthetic images, which represent our ground truth, are generated and converted to DICOM images with a resolution of 512 × 512 pixels^2^, 3 mm thickness, and 1.2 mm pixel size. Figure 5 shows a 3D virtual phantom covering a FOV of 500 × 450 × 300 mm^3^ and a corresponding two‐dimensional (2D) image. Our software is then used to calculate the distortion on these distorted synthetic images and the results are compared to the ground truth to assess the accuracy of our software.

**FIGURE 6:**
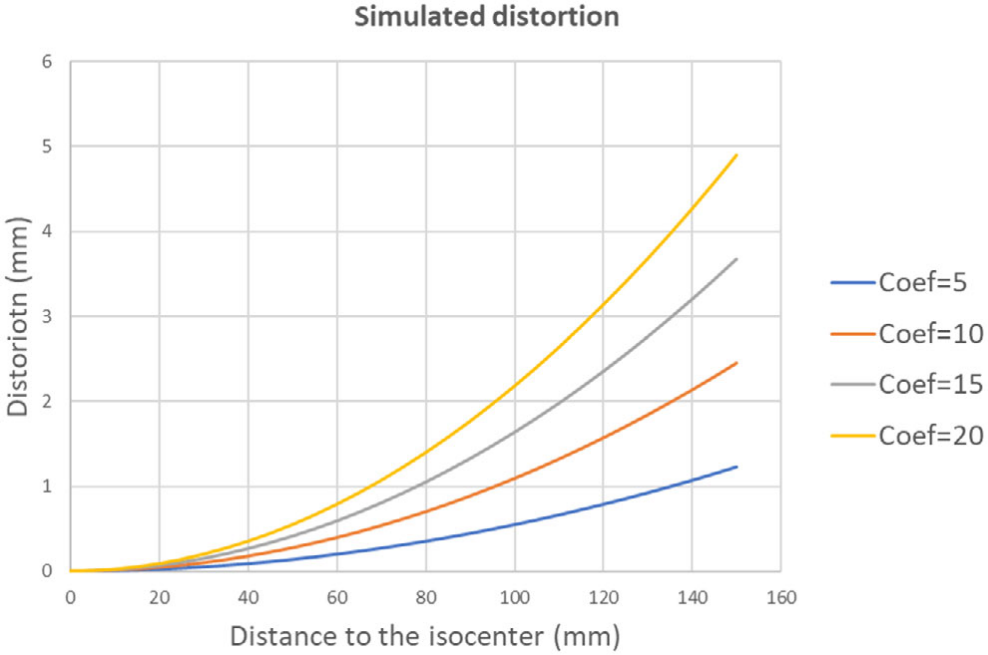
Four simulated distortion using different values of Coef

The amount of geometric distortion applied to the images is based on the following model:

(2) $$G\;=\;1-\mathrm{Coef}\times \frac{\frac{4}{3}\times d^{2}}{r^{2}},$$

where G is the distortion (mm), Coef is a coefficient that we will define, d (mm) is the pixel position with respect to the isocenter, and r is the gradient coil radius (mm). For each gradient, a series of distorted images have been generated by varying the value of Coef from 5 to 20 as shown in Figure 6.

## 3 RESULTS

### 3.1 Software validation

The application of our algorithms on the synthetic images enabled measuring the positions of the tubes within a sub‐pixel precision. Table 2 shows a comparison between the calculated distortion and the simulated “Ground truth” for different values of coefficient. These calculations were performed on images before and after applying a scaling factor of 2.

**TABLE 2:** Comparison of the calculated distortion with the simulated “ground truth” for different values of coefficient

		**Our software (mm)**		
**Coefficient**	**Ground truth (mm)**	**Scaling factor 1**	**Scaling factor 2**	**Radial distance (mm)**
5	0.03	0.1	0.2	0–50
10	0.06	0.4	0.3	
15	0.1	0.2	0.2	
20	0.13	0.9	0.4	
5	0.3	0.2	0.4	50–100
10	0.6	0.4	0.7	
15	0.9	0.7	0.9	
20	1.2	1.3	1	
5	0.8	0.7	0.9	100–150
10	1.7	1.5	1.5	
15	2.5	2.2	2.3	
20	3.4	3.8	3.2	
5	1.6	1.8	1.7	150–200
10	3.3	3.1	3.2	
15	5	5.2	5.2	
20	6.6	6.3	6.7	

Without the scaling factor, the mean errors in distortion were 0.3, 0.1, 0.2, and 0.2 for radial distances of 50, 100, 150, and 200 mm, respectively. After applying a scaling factor of 2 on the same images, the mean errors in distortion were 0.2, 0.1, 0.2, and 0.1 for radial distances of 50, 100, 150, and 200 mm respectively.

### 3.2 Phantom reproducibility

The error in the position of the tubes represented by the mean value of the geometric distortion was greater for the peripheral tubes and becomes smaller for the central tubes. An error of approx. ±0.2 mm is present for the tubes positioned more than 150 mm from the isocenter. The error drops to values lower than ±0.1 mm as the distance to isocenter becomes below 150 mm.

### 3.3 Geometric distortion

**FIGURE 7:**
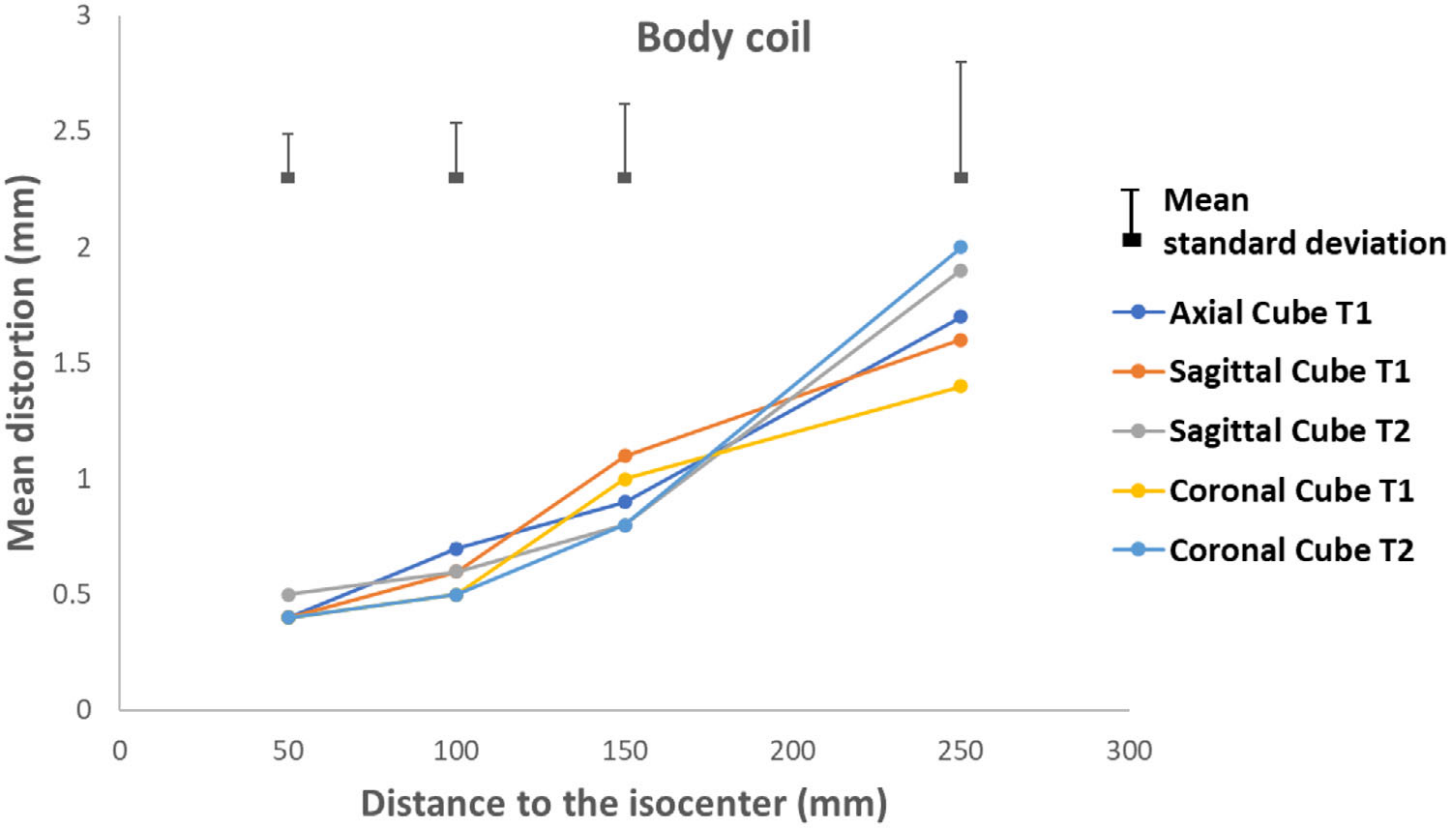
Geometric distortion for the body coil

**FIGURE 8:**
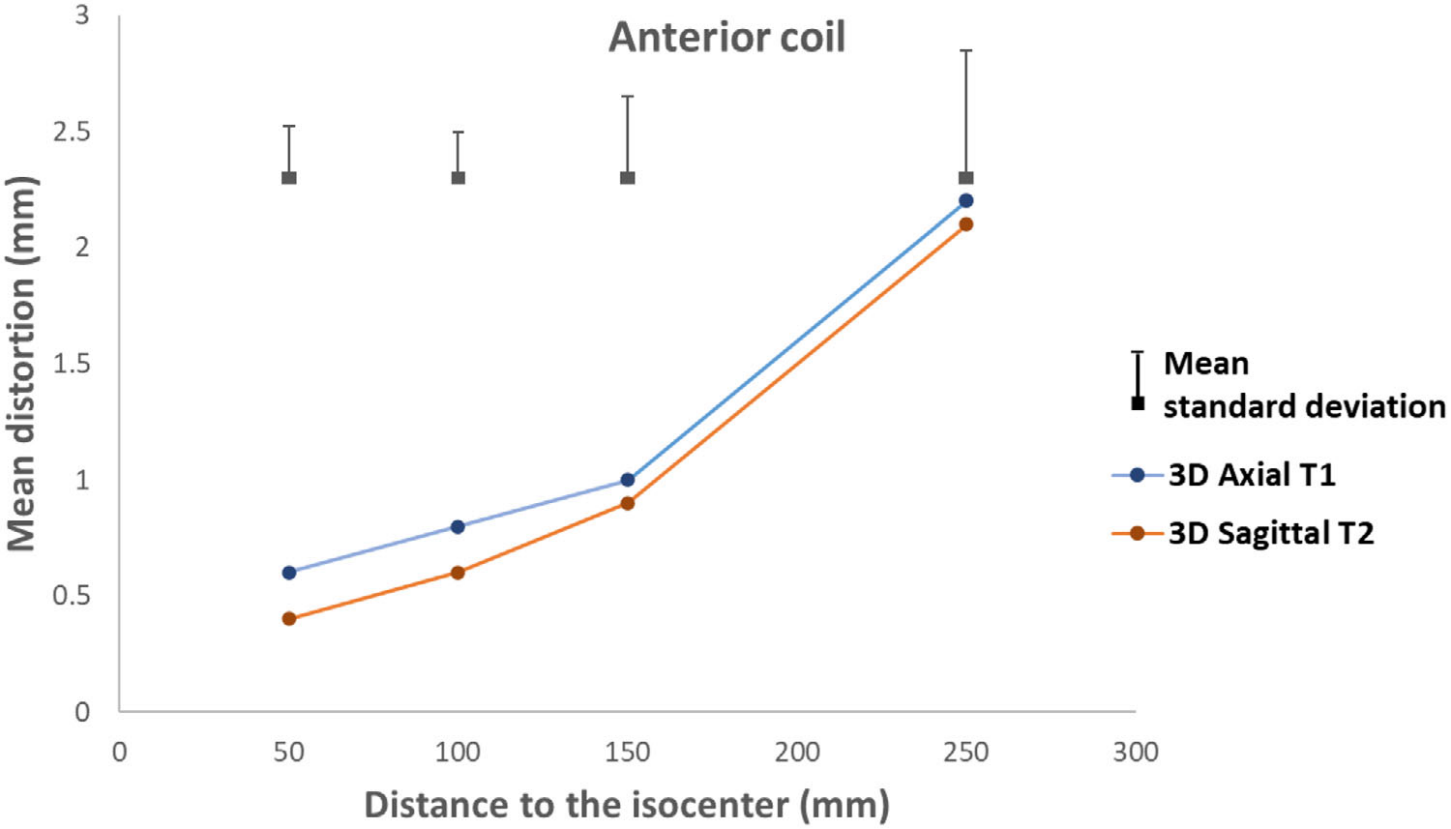
Geometric distortion for the anterior coil

**FIGURE 9:**
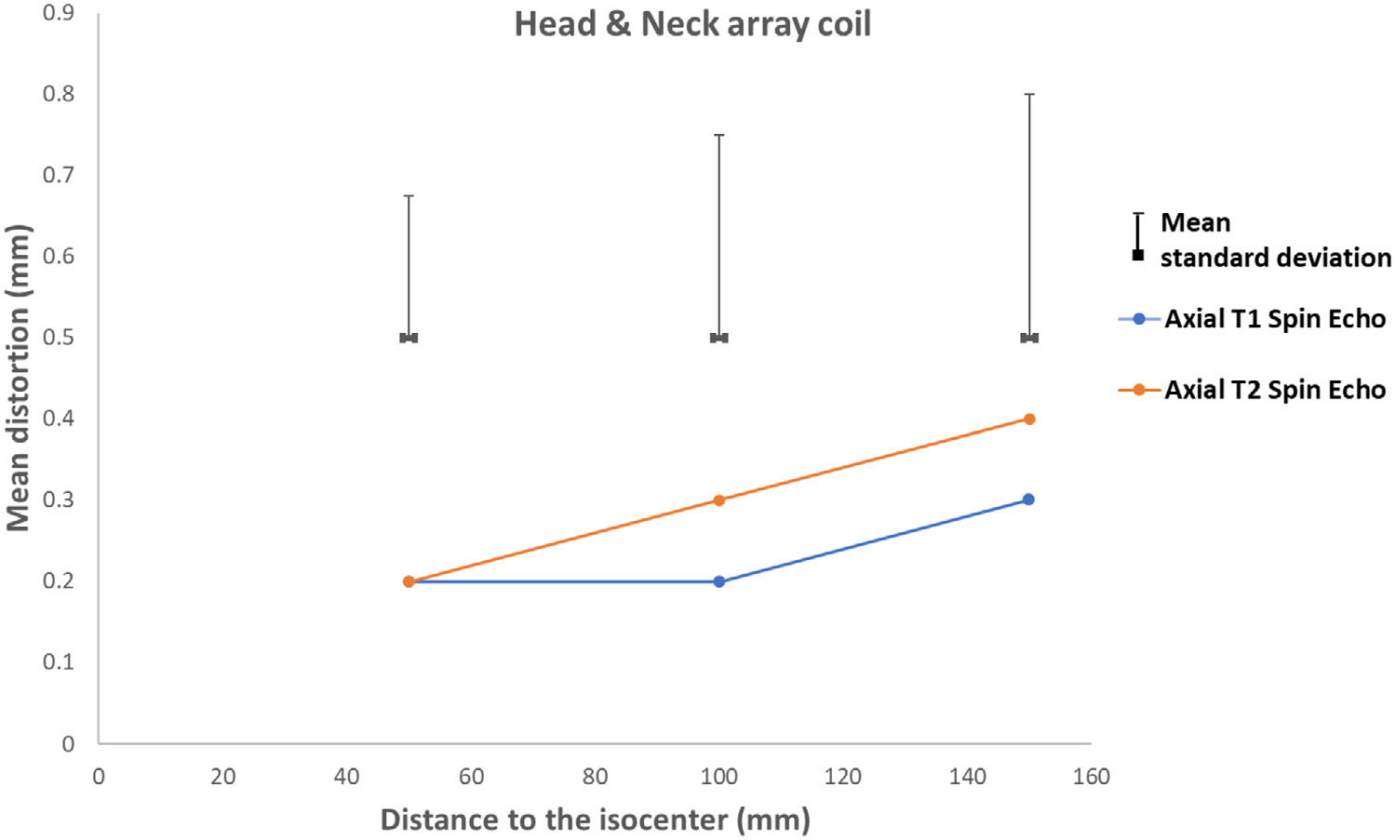
Geometric distortion for the head &and neck array coil

**FIGURE 10:**
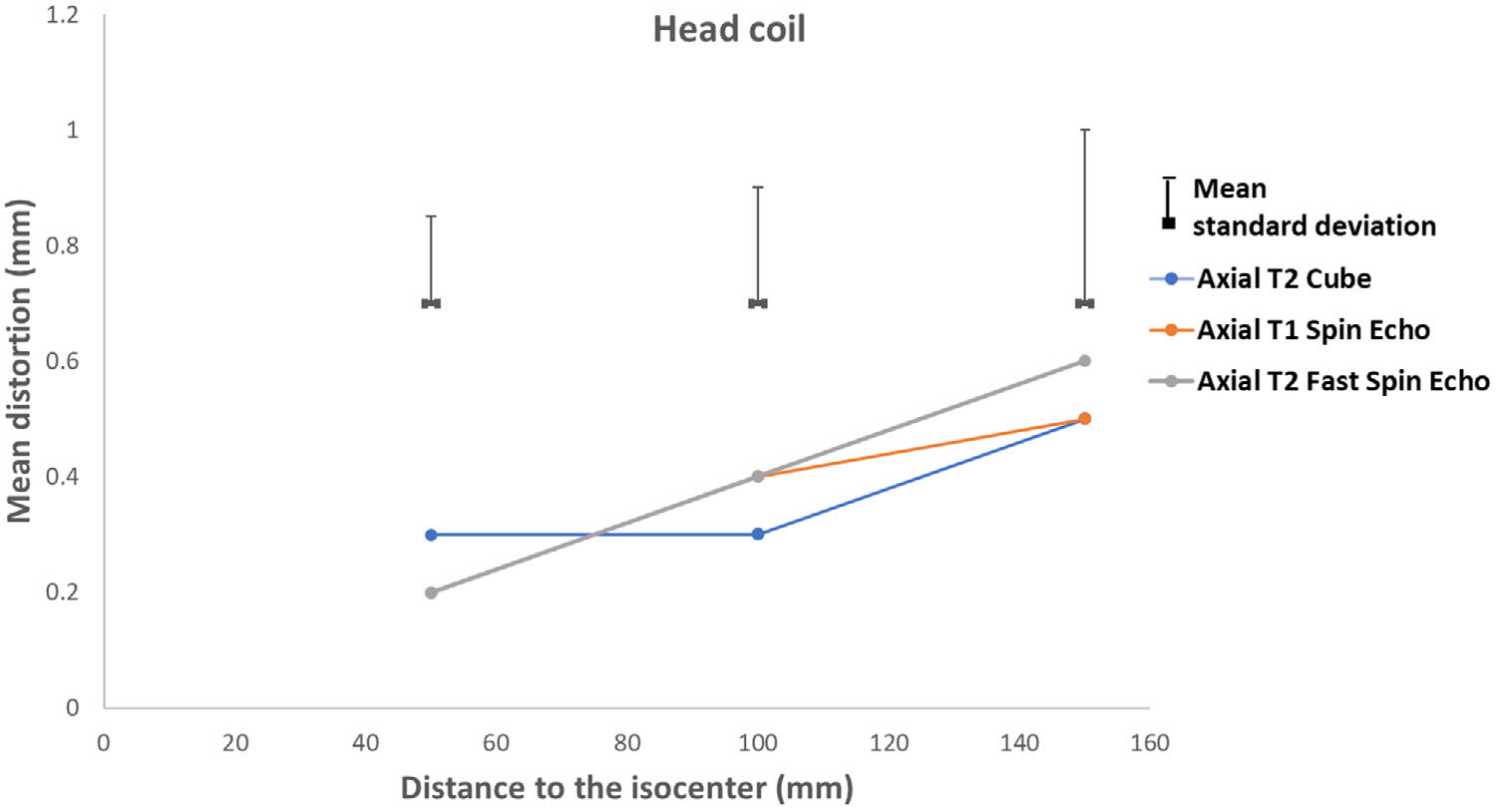
Geometric distortion for the head coil

Figures 7, 8, 9, 10 present the geometric distortion for the body coil, the anterior array coil, the head and neck array coil, and the head coil, respectively.

For the body coil, the mean distortion was 0.4, 0.6, 0.9, and 1.5 mm with mean standard deviations of 0.2, 0.2, 0.3, and 0.5 for radial distances of 50, 100, 150, and 250 mm, respectively.

For the anterior coil, the mean distortion was 0.5, 0.6, 1.1, and 1.7 mm with mean standard deviations of 0.22, 0.2, 0.4, and 0.5 for radial distances of 50, 100, 150, and 250 mm, respectively.

For the head coil, the mean distortion was 0.2, 0.2, and 0.4 mm with mean standard deviations of 0.15, 0.2, and 0.3 for radial distances of 50, 100, and 150 mm, respectively.

For the head and neck array coil, the mean distortion was 0.2, 0.4, 0.5, and 0.8 mm with mean standard deviations of 0.17, 0.25, and 0.3 for radial distances of 50, 100, and 150 mm, respectively.

## 4 DISCUSSION

The use of MRI to guide radiotherapy is partially limited by the existence of system‐related geometric inaccuracies caused mainly by the inhomogeneity in the main magnetic field and the nonlinearities of the gradient coils. Furthermore, there is a paucity of equipment and software needed to encompass the test of geometric distortion as part of routine clinical quality assurance (QA) of MRI image‐guided applications.

A considerable amount of work has been carried on the use of phantoms for assessing MRI geometric distortion.^11^, ^12^, ^13^, ^14^, ^15^, ^16^, ^17^, ^18^, ^19^ In this study, a new design based on 3D printed plastic blocks allowing the creation of modular phantoms has been proposed. This study is the latest to date in our effort to characterize the system‐related geometric distortion. In our first study,^11^ a water‐filled cylinder containing rods covering a FOV of 420 mm has been used while in the second study,^12^ we have used a 500 × 500 × 500 mm^3^ phantom composed of foam layers embedded with vitamin D capsules.

The design presented in this study presents many advantages over the designs presented in similar studies. The first advantage is represented by the modulability of the structure. By using plastic blocks clipped together, geometric distortion phantoms with adapted geometry can be constructed to fit any type of radio frequency (RF) coil. In this study, phantoms for measuring the geometric distortion of the body, the anterior array, the head and head and neck array coils were constructed. The average time to assemble a phantom from the blocks was 15 min. Furthermore, the fact that these blocks are clipped together makes the phantom robust and minimizes the uncertainty coming from the set‐up. Finally, these blocks are 3D printed with an average printing time of 30 min for each block. As such it can be easily produced or replaced in case of any damage.

The phantoms assembled in this study weight between 0.5 kg and 2 kg which makes it easy to manipulate and represents another important advantage compared to the water‐filled based phantoms that weight more than 20 kg.

As presented, rods/tubes were inserted in the plastic holes in order to the geometric distortion. The use of tubes presents a considerable advantage over points when measuring the geometric distortion in the plane perpendicular to these tubes. The acquisition procedure is made easier since these tubes cover the whole space perpendicular to the acquisition plane without any gap. As such, only few slices are required since it is impossible to miss the tubes during the acquisition and signal that come from the water inside the tubes will always be present in the images. For the phantoms using control points instead of tubes, a longer acquisition time is required since the whole 3D volume should be acquired before measuring the geometric distortion. In our example, for the body coil, 1 min 28 s were needed to acquire images for measuring the in‐plane distortion for the axial T2 spin echo sequence. The acquisition of the whole volume would have lasted 7 min 30 s in case a control point‐based phantom is used. The acquisition time needed for measuring the in‐plane geometric distortion for the head coil was reduced from 6 min 28 s to 2 min 16 s for the axial T1 spin echo. For all the coils, the use of tubes allowed a reduction of a minimum 20% in the acquisition time compared to the control point‐based phantoms. Therefore, in addition of allowing to accurately characterize the geometric distortion over a large FOV, the use of this phantom allows the reduction of the acquisition time. As such, this phantom will represent a valuable tool for the implementation of a simple, accurate, and fast daily/weekly procedure for characterizing MRI geometric distortion on several coils.

However, when using tubes, only the geometric distortion within the plane perpendicular to these tubes can be measured. To overcome this limitation and to allow measuring 3D geometric distortion, the tubes were distributed parallel to two planes. As such, in‐plane and through‐plane distortion can be measured for all acquisitions. The another advantage of using tubes is the possibility to easily replace the water with any desired liquid.

The software for automatic measurement of geometric distortion has been developed and presented. Regarding the software validation method, unlike other methods that use CT/MRI acquired images for validation purposes, our validation method is based on synthetic images. Synthetic images reproducing the exact geometry of the phantoms have been generated and well‐known geometric distortion has been introduced. The calculated distortion is then compared to the well‐known applied distortion in order to measure the accuracy of the software. This method eliminates uncertainties introduced by the acquisition and the image reconstruction processes.

The design presented in this work represents a valuable approach for the construction of phantoms aimed at the characterization of the MRI geometric distortion. The novelty of this work comes first from the use of 3D printed plastic blocks which hold glass tubes filled with any desired liquid. This approach allows assembling phantoms that can fit any RF coil with a setup uncertainty of less than 0.3 mm.

We are currently extending this work to create phantoms with different geometry. The water inside the tubes will also be replaced and contrast material will be injected in order to characterize the quality of correspondent MRI sequences. Finally, the phantoms and the software presented in this study will be part of a quality assurance program aimed at monitoring the image quality of MRI scanners used to the guide radiation therapy. It will also be used across our corporation for characterizing the geometric distortion of all our MRI scanners.

## 5 CONCLUSION

In this study, a novel design for a customizable MRI phantom for characterizing the system‐related geometric distortion has been proposed. This design is based on 3D printed plastic blocks allowing the creation of modular phantoms that can fit any RF coil. This solution meets all expectations in terms of having lightweight, customizable, and practical tool for measuring distortion in three dimensions.

These tools will be part of a quality assurance program aimed at monitoring the image quality of MRI scanners used to guide the radiation therapy.

## AUTHORS CONTRIBUTION

Tarraf Torfeh: Designed the study. Acquired and analyzed the data. Wrote the paper.

Rabih Hammoud: Contributed to the design of the study, data acquisition, and to the writing of the manuscript.

Satheesh Paloor: Contributed to the writing of the manuscript.

Yoganathan Arunachalam: Contributed to the writing of the manuscript.

Souha Aouadi: Contributed to the writing of the manuscript.

Noora Al‐Hammadi: Contributed to the design of the study and the writing of the manuscript.
